# Non-flooded riparian Amazon trees are a regionally significant methane source

**DOI:** 10.1098/rsta.2020.0446

**Published:** 2022-01-24

**Authors:** Vincent Gauci, Viviane Figueiredo, Nicola Gedney, Sunitha Rao Pangala, Tainá Stauffer, Graham P. Weedon, Alex Enrich-Prast

**Affiliations:** ^1^ Birmingham Institute of Forest Research, University of Birmingham, Edgbaston, Birmingham, UK; ^2^ School of Geography Earth and Environmental Science, University of Birmingham, Edgbaston, Birmingham, UK; ^3^ Multiuser Unit of Environmental Analysis, University Federal of Rio de Janeiro, Rio de Janeiro, Brazil; ^4^ Met Office Hadley Centre, JCHMR, Maclean Building, Benson Lane, Crowmarsh Gifford, Wallingford, Oxfordshire OX10 8BB, UK; ^5^ Lancaster Environment Centre, Lancaster University, Bailrigg, Lancaster LA1 4YQ, UK; ^6^ Biogas Research Center and Department of Thematic Studies – Environmental Change, Linköping University, Linkoping SE-581 83, Sweden

**Keywords:** methane, Amazon, floodplain, riparian, trees, soils

## Abstract

Inundation-adapted trees were recently established as the dominant egress pathway for soil-produced methane (CH_4_) in forested wetlands. This raises the possibility that CH_4_ produced deep within the soil column can vent to the atmosphere via tree roots even when the water table (WT) is below the surface. If correct, this would challenge modelling efforts where inundation often defines the spatial extent of ecosystem CH_4_ production and emission. Here, we examine CH_4_ exchange on tree, soil and aquatic surfaces in forest experiencing a dynamic WT at three floodplain locations spanning the Amazon basin at four hydrologically distinct times from April 2017 to January 2018. Tree stem emissions were orders of magnitude larger than from soil or aquatic surface emissions and exhibited a strong relationship to WT depth below the surface (less than 0). We estimate that Amazon riparian floodplain margins with a WT < 0 contribute 2.2–3.6 Tg CH_4_ yr^−1^ to the atmosphere in addition to inundated tree emissions of approximately 12.7–21.1 Tg CH_4_ yr^−1^. Applying our approach to all tropical wetland broad-leaf trees yields an estimated non-flooded floodplain tree flux of 6.4 Tg CH_4_ yr^−1^ which, at 17% of the flooded tropical tree flux of approximately 37.1 Tg CH_4_ yr^−1^, demonstrates the importance of these ecosystems in extending the effective CH_4_ emitting area beyond flooded lands.

This article is part of a discussion meeting issue 'Rising methane: is warming feeding warming? (part 2)'.

## 1. Introduction

Methane (CH_4_) is the second most important greenhouse gas and wetlands constitute the largest individual source emitting an estimated 102–200 Tg of CH_4_ to the troposphere each year [1]. Given their importance to the atmospheric CH_4_ budget, there is considerable effort devoted to quantifying wetland emissions and characterizing fluxes at the global scale. A principal characteristic of so called process-based or ‘bottom up’ models of CH_4_ emission from wetlands is that soils and sediments are inundated in order for CH_4_ to be produced and emitted [2]. These models are usually parameterized against flux measurements from only soil, aquatic or herbaceous surfaces [3,4]. However, there is a growing body of evidence demonstrating that trees can access and transmit CH_4_ from within the soil column of both temperate [5–7] and subtropical and tropical wetlands [8–10]. We recently reported that tree emissions dominate the CH_4_ budget of the Amazon basin, with trees within the pulsing hydrological system of the floodplain contributing around half of all methane from the region [11]. The presence of wetland-adapted trees as an important egress pathway presents a more pronounced vertical dimension to previously examined emission pathways both above and below the forest floor. This complicates approaches to quantifying emissions, but yields opportunities to consider new processes of CH_4_ source access, entrainment and evasion.

Tree roots penetrate down to around 6 m, and up to 18 m beneath the forest floor in broad-leaved tropical forest [12]. This raises the possibility that CH_4_ produced deep within the soil, which would normally be consumed by soil oxidation while diffusing to the surface [13], is instead entrained within wetland tree roots that access anaerobic soil microsites. The root-entrained CH_4_ is then transported to the surface via the tree's vascular system and emitted from the stem surfaces. This process has been identified in a number of studies where CH_4_ fluxes were observed to correlate with WT depth below the soil surface at relatively shallow depths [10,14,15]. Furthermore, in ostensibly dry upland soils, trees emit CH_4_ from their stem bases [16,17].

Seasonal flooding along the Amazon river and its tributaries is followed by prolonged periods of low water-table and, on occasion, drought with the period of low water varying across the Amazon [18]. We explore the possibility that trees, adapted to inundation through internal architecture facilitating root aeration [19], may be emitting CH_4_ sourced from below the soil surface during dry conditions when the WT is below the soil surface. This opens the possibility that previous efforts to characterize CH_4_ fluxes from trees in the Amazon floodplain [11] during a single high water event may have missed a significant source of CH_4_. Gedney *et al.* [20] sought to characterize this below-ground WT < 0 contribution to emissions by extending a process-based CH_4_ emission model to incorporate a simple parameterization for a tree-mediated flux from saturated soils. However, due to a lack of available measurements from trees with a WT below the surface, they assumed a relative tree flux dependency on both WT depth and root density distributions, which they combined to produce a global wetland flux consistent with best estimates. Field measurements are therefore required to evaluate this idea fully. To better characterize the Amazon CH_4_ budget, we measured CH_4_ fluxes across four seasonal intervals for three representative Central Amazonian floodplain forests. This involved repeated visits to the same individual trees experiencing inundation and, by turn, low water.

## 2. CH_4_ flux measurements

**Figure 1.**
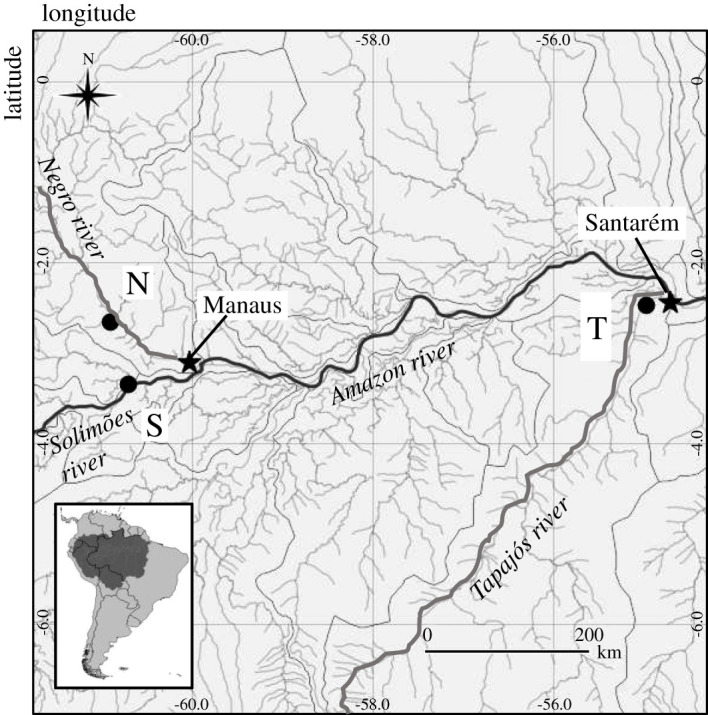
Map showing the locations of the three sampling sites within the floodplains of Solimões (S; white water) Negro (N; black water) and Tapajós (T; white water) rivers.

We established three plots spanning a topographic gradient from the water's edge, within seasonally flooded forests within the 1.77 million km^2^ reference quadrants of the central Amazon basin [21]. The temporary plots (60 × 60 m) were set up within the floodplains of three major rivers of the Amazon (figure 1): the Negro river (black water), Solimões river (white water) and Tapajós river (clear water). We quantified CH_4_ fluxes from a total of 108 trees (36 across each plot) at vertical intervals above the forest floor/aquatic surface across four fieldwork campaigns: rising water (campaign 1; April 2017), peak water (campaign 2; July 2017), receding water (campaign 3; October 2017) and low WT (campaign 4; January 2018). We further quantified CH_4_ fluxes on soil and aquatic surfaces within each plot.

Each plot was divided into three distinct hydrological zones: (1) wet zone—closest to the river channel/lake (2) an intermediate zone and (3) dry zone—furthest away from the water source (electronic supplementary material, table S1). The plot in Solimões was an exception to this, as all three zones were of the same elevation, hence experienced similar WT depths (electronic supplementary material, table S1). In each of these plots, intensive field campaigns were carried out at the four distinct hydrological time points. At the plot level, within each of the hydrological zones (60 × 20 m), stem CH_4_ fluxes were measured from 12 trees, resulting in a total of 36 trees measured per plot, with the same trees measured across all four campaigns. Stem CH_4_ fluxes were measured at three points between 20 and 120 cm above the soil or aquatic surface (20–50 cm, 55–85 cm and 90–120 cm), depending on whether the WT level was above or below the soil surface. WT depths when above the water surface were measured using a marked, weighted cord at two locations around the tree under investigation and averaged to obtain a mean WT position. For the dry zone, the ground elevation, distance from the water body and water levels within the plot and river were recorded to calculate the relative WT levels below the soil surface. A similar approach was used during the receding and low water field campaigns when the WT was below the soil surface, where the WT level in the adjoining lake or river along with the elevation at each of the trees were measured and their distance from the water body was used to calculate the WT height.

Tree stem CH_4_ emissions (*n* = 432 per plot across all four campaigns) were measured using static chambers as described in Siegenthaler *et al.* [22] and Pangala *et al.* [11]. During inundated periods (campaigns 1 and 2), CH_4_ emissions from the aquatic surfaces within each plot (*n* *=* 168 per plot across the two campaigns) were measured using floating chambers as described in Bastviken *et al.* [23] and Pangala *et al.* [11]. There were no aquatic fluxes measured in campaigns 3 and 4 as the WT was below the soil surface. Soil CH_4_ fluxes were measured using cylindrical static chambers as described in Pangala *et al.* [11]; five chambers were placed within each hydrological zone where the WT was below the soil surface and as and when the WT receded additional soil chambers were installed to measure the soil flux. Therefore, soil CH_4_ flux measurements equated to: (a) campaign 1: *n* = 5 in Tapajós and Negro plot, no soil flux was measured in the Solimões plot as the entire plot was flooded, (b) campaign 2: *n* = 5 in the Negro plot, *n* = 10 in Tapajós plot as the water receded more quickly at this site relative to others and no soil flux measurement in Solimões plot and (c) campaigns 3 and 4: *n* = 15 in each plot.

As and when it was possible, stem and soil CH_4_ fluxes were measured by cavity-enhanced absorption spectroscopy (LGR ultraportable greenhouse gas analyser, ABB, Canada) as described in Pangala *et al*. [11]; however, when it rained or in the absence of the instrument due to repair, gas samples were extracted from the tree at *T* *=* 1, 5, 10, 15 and 20 min, *T* = 0, 10, 20, 30 and 40 min from soil chambers and *T* = 0 and 24 h from aquatic chambers. Gas samples extracted using gas tight syringes from tree stem, soil and aquatic chambers were transferred to 12 ml Labco glass vials (Labco Ltd. Ceredigion, UK) and analysed for CH_4_ using modified cavity ring-down laser spectroscopy [11,24]. CH_4_ fluxes are expressed per unit area enclosed by the chamber on the tree, soil or aquatic surface, respectively and therefore reported as mg m^−2^ h^−1^ corresponding to mg m^−2^ soil h^−1^ for soil fluxes, mg m^−2^ stem h^−1^ for tree stem fluxes and mg m^−2^ aquatic h^−1^ for aquatic fluxes (table 1).

**Table 1.** Summary table of CH_4_ fluxes for each measured surface across all four campaigns. Large standard deviations reflect broad topographical gradients spanned within each plot and known species dependency on fluxes e.g. [8]. *n* = 36 for tree flux measurements at each plot per campaign. Sixty-four aquatic fluxes were made at each location during the first two campaigns only and soil flux measurement n were as follows: campaign 1: *n* = 5 in the Tapajós and Negro plots; campaign 2: *n* = 5 in the Negro plot, *n* = 10 in the Tapajós plot and campaigns 3 and 4: *n* = 15 at each plot.

		CH_4­_ flux mg m^−2^ h^−1^ (± s.d.)			
surface	location	Apr 2017	July 2017	Oct 2017	Jan 2018
tree stem (20–50 cm)		s.d.	s.d.	s.d.	s.d.
	Solimões	61.9 ± 68.2	78.9 ± 52.3	8.19 ± 15.7	0.0128 ± 0.042
	Negro	38.2 ± 53.3	55.8 ± 50.5	5.18 ± 11.8	0.0052 ± 0.015
	Tapajós	69.7 ± 75.8	9.6 ± 20.2	5.94 ± 11.7	−0.0045 ± 0.007
aquatic surface					
	Solimões	1.84 ± 1.85	2.33 ± 18.7	dry	dry
	Negro	0.49 ± 0.48	0.77 ± 0.73	dry	dry
	Tapajós	1.68 ± 2.26	1.64 ± 1.47	dry	dry
soil surface					
	Solimões	flooded	flooded	−0.022 ± 0.0435	0.0092 ± 0.0158
	Negro	0.027 ± 0.165	0.049 ± 0.9	−0.039 ± 0.0262	−0.0015 ± 0.0123
	Tapajós	−0.013 ± 0.103	−0.008 ± 0.031	−0.043 ± 0.0403	−0.0094 ± 0.0166

**Figure 2.**
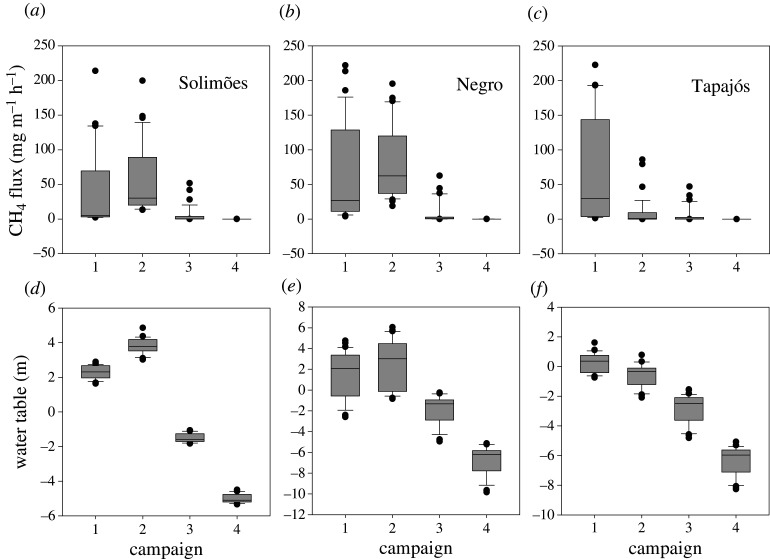
Box and whisker plots of seasonal changes in tree CH_4_ flux measured at 20–50 cm above the forest floor (either soil or water depending on the state of flood) for the three study plots in each catchment (*a*–*c*). Figure (*d*–*f*) demonstrates the corresponding water table for each location at each seasonal time point. Campaigns 1 through 4 were carried out, respectively, during rising (campaign 1; April 2017), peak (campaign 2; July 2017), receding (campaign 3; October 2017) and low water table conditions (campaign 4; January 2018). Error bars indicate the 10th and 90th percentiles.

The four selected sampling season intervals were successful in representing the full hydrological range experienced within these study tributaries in the Amazon basin (electronic supplementary material, table S1). Due to topography along each monitoring transect, Solimões had the narrowest within site water-table range, with Negro the largest. Both Solimões and Negro experienced a peak inundation in campaign 2 (July 2017) and subsequent declining water level/table with both sites experiencing below surface WT in the October 2017 and January 2018 (figure 2; electronic supplementary material, table S1). By contrast, Tapajós already had a peaking WT in campaign 1 (April 2017) with a soil surface that was only partially submerged during this peak flood. Thereafter the WT declined with WT reaching up to approximately 8 m below the soils surface.

CH_4_ fluxes at the lowermost sampling position on each tree (20–50 cm above the forest floor/water surface) during rising and peak water for all three catchments were comparable to emissions observed by Pangala *et al.* [11] of approximately 40–80 mg CH_4_ m^−2^ h^−1^ (table 1) and, as in that study, stem fluxes declined with tree height. We only observed these large tree stem base fluxes during the first sampling campaign at Tapajós (table 1 and figure 2). CH_4_ fluxes during these rising and peak water periods were the largest recorded from any surface with aquatic and soil surface fluxes orders of magnitude smaller than those from trees (table 1). CH_4_ emissions declined substantially from tree stems when the water-table fell to below the soil surface in campaigns 3 and 4 (also 2 in Tapajós). While we tended to observe emissions from these sites in October and January (campaigns 3 and 4) when the WT was below the surface, the range in tree fluxes observed during the driest sampling (January) (when the WT at all sites was lower than 5 m below the soils surface (electronic supplementary material, table S1)) suggests that trees no longer accessing CH_4_-rich pore waters possess the capacity to switch function from CH_4_ emission to uptake.

## 3. From local to Amazon basin and tropical upscaling using JULES

To scale our findings, we first sought to establish total tree fluxes. We measured tree height, diameter at breast height (DBH), stem diameter at 10 cm intervals in the bottom-most 150 cm of exposed tree stem, along with the basal diameter for all trees within each plot. This allowed the total exposed tree surface area to be estimated for each plot across all four campaigns. The stem diameters measured between stem sampling positions of 20 and 120 cm above the forest floor/water surface, the stem surface area was calculated by considering each tree as a truncated cone that was divided into 30 cm sections [8]. The relationship established between stem position above the forest floor/water surface and corresponding stem diameter measured between 10 and 150 cm was applied to the entire length of the tree which allowed total tree flux to be estimated as described in Pangala *et al.* [11]. Figure 2 clearly shows that the tree mediated CH_4_ flux (CH_4tree_) is dependent on WT when it is below the soil surface.

To uncover any empirical relationships, we regressed total tree flux against WT depth (*z*_WT_). If we assume that rooting density decreases approximately exponentially with soil depth [25], then the relative number of roots beneath a specified soil depth would also decrease exponentially with depth. The tree flux originating from the saturated zone below the soil surface may therefore be described as a function of WT depth below the surface:

$$\text{C}{\text{H}}_{4\mathrm{tree}}=\text{exp}(C).\text{exp }(M.z_{\mathrm{WT}})\;\text{for}\;z_{\mathrm{WT}}<0,$$

where *C* and *M* are tuneable parameters. This is equivalent to

3.1 $$\text{ln}(\text{C}{\text{H}}_{4\mathrm{tree}})=M.z_{\mathrm{WT}}+C\;\text{for}\;z_{\mathrm{WT}}<0.$$

**Figure 3.**
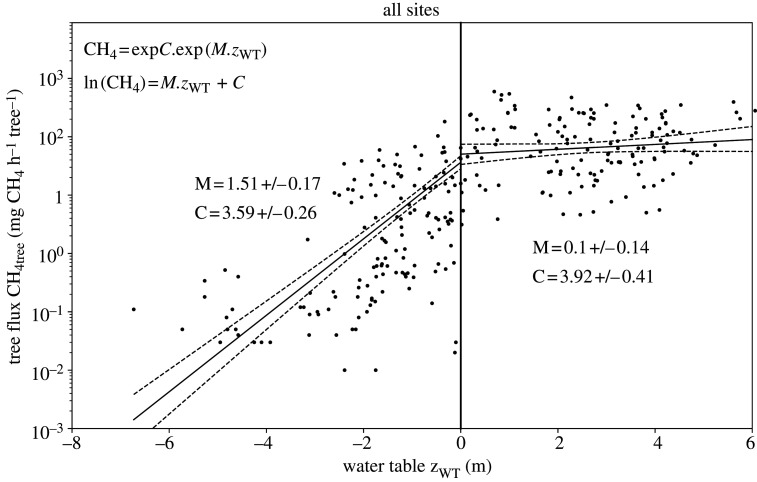
The dependence of total tree flux on water table. Measurements are shown as black dots. *z*_WT_〈0, and〉0 refer to water table below and above the soil surface, respectively. Linear regressions of ln(CH_4tree_) = *M*.*z*_WT_ + *C* (equation (3.1)) are applied to all site data when the water table is at or above the soil surface (*z*_WT_ ≥ 0), and applied to all site data but limiting *z*_WT_ in equation (3.1) to be no higher than the soil surface (i.e. *z*_WT_ ≤ 0) – see text for details. Mean and 95% confidence intervals are shown with solid and dashed lines, respectively.

If roots are the primary facilitating factor then it might also be expected that, for WTs above the surface, the tree flux is independent of WT height. To confirm this, first we apply a linear regression to the measured WT and natural logarithm of tree flux (equation (3.1); figure 3) using data from all sites and campaigns when the WT is above the soil surface. This yields a negligible gradient *M*, which is not statistically significantly different from zero with *M* = 0.10 ± 0.14 (where the ± value is 95% confidence interval from hence forth, unless stated otherwise). Utilizing this result we apply a linear regression to *all* the data, but limit *z*_WT_ in equation (3.1) to be no higher than the soil surface (i.e. *z*_WT_ ≤ 0). This gives a gradient *M* of 1.51 ± 0.17 and implies roughly a 150% increase in flux per 1 m increase in *z*_WT_ (*d*[CH_4tree_]/CH_4tree_ = *Mdz*_WT_).

Results of our regression analysis (figure 3) clearly demonstrate that the tree-mediated CH_4_ flux (CH_4tree_) is strongly dependent on WT depth beneath the soil surface. This is consistent with tree roots playing a facilitating role in the CH_4_ transfer.

To investigate the significance of this at the regional scale, we use an optimized version of the land surface model JULES ([25,26], electronic supplementary material). As well as simulating the flux of water through shallow soil layers, JULES includes a simple groundwater model which can simulate WT depth: a requirement for the regional WT dependent tree flux estimates. Although JULES lacks the detailed inundation modelling in hydrological models specifically calibrated to the Amazon (e.g. [27,28]) it is a global scheme, enabling us to estimate the magnitude of this flux over both the Amazon basin and all tropical forests. JULES combines modelled grid box mean WT depth with sub-grid topographic distribution to produce the sub-grid, WT distribution *fw*’(*z*) (see electronic supplementary material for details). The flux dependence on depth is combined with *fw*’(*z*) and tree cover to scale up the fluxes initially over the entire Amazonian forest.

Nine surface tile fractions are specified in each JULES grid-box *i* [29]. These are produced by remapping and reclassifying land cover maps from the International Geosphere-Biosphere Programme (see [29] for details). These tiles include an open water tile and five plant functional types, with broadleaf trees dominant in tropical forests. To extrapolate up from local tree density measurements from Pangala *et al.* [11] (electronic supplementary material), we must equate basal tree area with prescribed broadleaf tree fractional cover *f*_blt_ used in JULES. In the classification scheme used in JULES evergreen broadleaf forest *f*_blt_ is set as 0.85 [29]. The average tree densities measured for the Tapajós, Negro and Solimões sites is *ρ*(TNS) = 0.153 trees m^−2^. At low water, none of the sites in this study contain any open water, so we assume that the sites can be classified entirely as evergreen broadleaf forest. We therefore set the JULES broadleaf tree fraction from these sites *f*_b__lt_(TNS) to 0.85. We can then estimate the mean tree density for each grid box ‘i’ using the ratio of the grid box and site broad leaf tree fractions:

$$\rho (i)=\frac{f_{\mathrm{blt}}(i)}{f_{\mathrm{blt}}(\text{TNS})}\rho (\text{TNS}).$$

In each grid box, the sub-grid WT distribution *fw*^′^(*z*) is combined with the flux at WT depth *z*_WT_ and integrated. This is scaled by the tree density to give the total ‘sub-surface’ *F*_sub_(*i*) (i.e. when WT is below the surface) and surface *F*_sfc_(*i*) (WT is at or above the surface) tree fluxes (mgCH_4_ m^−2^ h^−1^):

3.2 $$F_{\text{sub}}(i)=\frac{\rho (\text{TNS})}{f_{\mathrm{blt}}(\text{TNS})}.f_{\mathrm{blt}}(i).\exp(C)\int_{-\infty }^{0}fw^{\prime }(i,z_{\mathrm{WT}}).\text{exp }(M.z_{\mathrm{WT}})\;\text{d}z_{\mathrm{WT}}$$

and

3.3 $$F_{\mathrm{sfc}}(i)=\frac{\rho (\text{TNS})}{f_{\text{blt}}(\text{TNS})}.f_{\mathrm{blt}}(i).\text{exp}(C)f_{s}(i),$$

where the inundation fraction is the integral over where the WT is above the surface: $f_{s}(i)=\int_{0}^{\infty }fw^{\prime }(i,z_{\text{WT}})\;\text{d}z_{\text{WT}}$. The total Amazon fluxes are calculated by multiplying each grid box flux by the grid box area and then summing over the entire basin.

**Table 2.** Amazon tree fluxes (Tg CH_4_ yr^−1^) averaged between years 2000 and 2009. Estimates are given for different JULES hydrology tuning parameter *f*exp values (electronic supplementary material). *F*_sfc_ and *F*_sub_ are the inundated (WT at or above the soil surface) and riparian (WT below the soil surface) tree fluxes, respectively. *F*_tot_ is the total tree flux (*F*_sfc_ + *F*_sub_). ‘CI’ represents results when using the 95% confidence intervals in tree flux fit, with gradients and intercepts: *M* = 1.51 + 0.17, *C* = 3.65–0.26 and *M* = 1.51–0.17, *C* = 3.59 + 0.26. Similarly, ‘s.d.’ represents the standard deviation in the fit.

tuning parameter	*F*_sub_	*F*_sfc_	*F*_tot_ = *F*_sub_ + *F*_sfc_	%*F*_sub_/*F*_tot_
*f*exp = 1 (ctl)	2.78	16.35	19.14	14.5
*f*exp = 1 lower CI	2.16	12.66	14.83	14.6
*f*exp = 1 upper CI	3.58	21.13	24.71	14.5
*f*exp = 1 − s.d.	2.45	14.36	16.81	14.6
*f*exp = 1 + s.d.	3.16	18.63	21.79	14.5
*f*exp = 0.5	2.37	13.94	16.31	14.5
*f*exp = 0.75	2.63	15.47	18.10	14.5
*f*exp = 1.5	2.99	17.52	20.52	14.6
*f*exp = 2.0	3.19	18.46	21.64	14.7

To scale up over the entire basin JULES is run at 0.5° resolution and forced off-line with observed meteorology [30] for the time period 2000–2009. The vegetation and soil properties are based on those described in [29], but with a modification to allow for better tropical soils representation (electronic supplementary material). JULES, with a modified version of hydrology (electronic supplementary material), is optimized (using tuning parameter *f*exp) to produce the best overall simulations of inundation and WT depths, as well as successfully simulating river discharge (electronic supplementary material). *f*exp = 1 (ctl) simulates the overall observed spatial distribution of inundation (electronic supplementary material). It produces total Amazon inundated tree areas of 2.54 × 10^11^ m^2^ and 2.88 × 10^11^ m^2^ averaged over low water 1995 and high water 1996, and 2000–2009, respectively. These are comparable to the mean estimated using the Hess *et al*. [31] (electronic supplementary material, 1.61–2.63 × 10^11^ m^2^) and WAD2M [32] (2.78 × 10^11^ m^2^) data from the same respective time periods (see electronic supplementary material for details).

The simulated WT measurement errors for the default JULES version are comparable to the Fan & Miguez-Macho [33] groundwater model (electronic supplementary material). Other *f*exp parameter values produce slightly lower WT errors (but worse inundation extents), so we consider these in sensitivity studies (table 2).

Our best estimate modelled total tree flux for the region is 19.1 Tg CH_4_ yr^−1^ (table 2, ctl), with a range of 16.8–21.8 Tg CH_4_ yr^−1^ allowing for the standard deviation of the regression fit. This modelled total Amazon tree flux is consistent with the Pangala *et al.* [11] estimate range of 13.3–23.7 Tg CH_4_ yr^−1^ based on flooded area observations. It is also consistent with the modelled estimates from Gedney *et al*. [20] (12.3–27.0 Tg CH_4_ yr^−1^). We estimate modelled tree emissions from areas with a WT at or above the surface is 16.4 (±95% CI 12.7–21.1) Tg CH_4_ yr^−1^. Our new observation-based approach of considering trees with a WT < 0 across seasonal WT fluctuations within the Amazon pulsing system results in an additional CH_4_ emission of 2.8 (±95% CI 2.2–3.6) Tg CH_4_ yr^−1^ from trees with a WT below the soil surface. This is about 15% of the total modelled tree flux and demonstrates the need to include tree emitted CH_4_ from floodplains where the soil surface is not inundated but has a near-surface WT extending as deep as approximately 7 m beneath the soil surface.

We extend this approach by utilizing the inundation and water tables simulated by JULES over the tropical regions defined as tropical S America, tropical Asia and tropical Africa in TRANSCOM [34]. Over all broadleaf tropical forests, this gives a total additional modelled sub-surface WT tree flux of approximately 6.4 Tg CH_4_ yr^−1^ and total tropical tree flux of 43.5 Tg CH_4_ yr^−1^, i.e. floodplain riparian trees that are not inundated emit approximately 15% of total tree CH_4_ flux and approximately 17% of the flooded wetland tree flux.

There are many uncertainties in this estimate, however. These include both limitations in the JULES model and lack of observations. JULES is designed so it can run globally, enabling it to produce regional and global flux estimates. It does not include some of the detailed modelling and calibration of flooding in hydrological models. There is limited availability of datasets of deeper sub-surface hydrological properties, as well as sparse measurements of WT depths in this region also limiting modelling capability. There are also uncertainties in inundation extent estimates [35]. The process of extrapolation from plot measurements to regional scale implicitly assumes that these plots are representative of the region. Thus there are many challenges which limit the tree flux estimations.

In spite of the uncertainties, these first estimates demonstrate that the tree flux, both from the riparian zone and the surface flooded area, is an important contributor to global tropical estimates. For example, the standard wetland CH_4_ formulation of JULES (which does not explicitly represent tree fluxes) estimates a total tropical emission of approximately 96 Tg CH_4_ yr^−1^. Our estimate of 43.5 Tg CH_4_ yr^−1^ emitted from both tropical riparian (6.4 Tg) and wetland (37.1 Tg) trees is nearly half of this total tropical wetland CH_4_ emission estimate. This is similar in proportion to the initial estimate of tree contributions to the Amazon CH_4_ budget [11] with trees representing a quarter of the upper estimate for the global wetland CH_4_ source [1].

## 4. Conclusion

Our results demonstrate a clear WT dependency on the CH_4_ emitting function of trees within the pulsing river floodplains of the Amazon basin. Among the different CH_4_ emission pathways, wetland adapted trees persisted in providing the largest emissions throughout periods of rising, peak and declining water-table in the first three measurement campaigns. Thereafter, in the final measurement campaign, emissions diminished to levels that were negligible in comparison to those measured under previous campaigns such that at the driest site, Tapajós, we observed trees taking up CH_4_ at a rate similar to those observed in soils.

We produced a regression model of the response of tree emissions to a varying WT that demonstrated a negligible response to increasing flood level above the soil surface but a clear dependence of ‘whole tree’ CH_4_ emissions on WT below the soil surface. We applied this relationship within JULES to scale our findings to the entire Amazon basin and to all tropical forests and found close agreement with the observation-based estimates reported by Pangala *et al.* [11]. We further found that approximately 15% of all tropical broadleaf tree CH_4_ emissions are emitted from trees with a WT that was below the soil surface. This amounted to an additional approximately 3 Tg of CH_4_ emissions within the Amazon basin and an additional approximately 6 Tg CH_4_ yr^−1^ for all broadleaf tropical forests. Our findings demonstrate the clear need to use approaches that capture, not only flooded tree flux but also this soil-derived, tree-mediated source of ‘non-wetland’ but anaerobically derived CH_4­_ from riparian trees when estimating global emissions.

## Data accessibility

All measurement data will be made available via the Centre for Environmental Data Analysis along with data from other MOYA contributors. The data are provided in electronic supplementary material [36].
